# Guanosine contributes to rapid purinergic regulation of dopamine during ischemia

**DOI:** 10.1007/s11302-026-10153-7

**Published:** 2026-04-20

**Authors:** Moriah E. Weese-Myers, Alaa S. Abdelrazeq Hassan, Ashley E. Ross

**Affiliations:** Department of Chemistry, University of Cincinnati, 312 College Dr. 404 Crosley Tower, Cincinnati, OH 45221-0172 USA

**Keywords:** Guanosine, Dopamine, Ischemia, Fast-scan cyclic voltammetry

## Abstract

Purinergic modulation of neurotransmission is a primary strategy employed in rapid neuroprotection during and after ischemic stroke. While the contributions of adenosine and ATP to the purinome are well-documented, guanosine-based regulatory activity has remained under-investigated. In the ischemia-vulnerable CA1 region of the hippocampus, dopamine serves as both a neurotransmitter involved in long-term potentiation during memory acquisition and as a regulator focused on modulating glutamate activity. Dopamine has been suggested as a potential protective force against glutamate-induced excitotoxicity in early ischemia. Here, we investigate changes in dopamine signaling during ischemia and demonstrate that guanosine administration during injury has an immediate protective effect. We observe rapid suppression of dopamine release following ischemic insult that is completely restored with administration of a low micromolar dose of guanosine. Changes in dopamine signaling and short-term mRNA production further suggest that guanosine’s rapid regulatory role may be partially achieved through the adenosine A1 and A2a receptors and that it facilitates dopamine reuptake through the dopamine transporter DAT. We present the first evidence that guanosine plays a rapid regulatory role in dopamine neurotransmission and that it may function as a broad facilitator of neurotransmitter reuptake, diversifying our understanding of the purinome in ischemia.

**Supplementary Information:**

The online version contains supplementary material available at 10.1007/s11302-026-10153-7.

## Introduction

Ischemic stroke is natively an acute pathology. Oxidative stress occurs within minutes of blood flow attenuation, with excitotoxic neurotransmitter accumulation and prolonged neuron depolarization following soon after. Damage can quickly spread beyond the initial point of injury as neurons become subject to spreading depolarization, dysregulating wide swaths of cells and causing lasting damage to surrounding regions. This problem is compounded in the particularly vulnerable CA1 region of the hippocampus [1, 2], which is subject to early and severe excitotoxicity during ischemic events [3]. Lasting CA1 injury can be debilitating because of the region’s role in spatial learning and memory [4, 5]. Consequently, the brain requires fast, nuanced neuroprotective strategies that can respond immediately to ischemic assault on a molecular and cellular level [6]. In the CA1, glutamate-based excitoxicity is regulated by a host of neuromodulators, including dopamine in a dual role as a neurotransmitter and regulator [7–10]. Purines have further emerged as essential neuroprotectors in ischemia; although previous work has focused mostly on ATP and adenosine, guanosine-based contributions have become an increasingly targeted topic of interest [11–14]. Here, we investigate the role of guanosine in correcting dopamine dysregulation during severe ischemia and establish early and immediate impacts on dopamine release and reuptake.

Dopamine (DA) is a neurotransmitter and neuroregulatory molecule that plays a nuanced role in CA1-based memory development [15–17]. Dopaminergic neurons from the ventral tegmental area (VTA) innervate the *stratum lacunosum moleculare* (SLM) as part of the glutamate-dopamine circuit between the hippocampus and midbrain [9, 18]. Both D1-like and D2-like receptors are expressed throughout the SLM and play a modulatory role on both glutamatergic pyramidal neurons and GABAergic interneurons [17, 19]. Reports on DA’s impact during ischemia are sparse, and both protective and injurious effects have been observed [20, 21]. Dopamine has previously been shown to exert a suppressive effect on glutamate through suppression of NMDAR activity [17, 22], thus making it a prime target for investigation as part of a cohesive neuroprotective strategy for mitigating glutamate-based excitotoxicity in ischemia.

Guanosine (GN) is a ubiquitous purinergic neuromodulator with fast neuroprotective activity during ischemia [14, 23]. While less well characterized than its sister nucleoside adenosine, GN’s role in the CA1 as a glutamate regulator is well established. It facilitates clearance of glutamate from the extracellular space, mitigating excitotoxicity, and has been suggested to antagonize excitatory NMDA receptors responsible for cell depolarization [14, 24–27]. Administration of GN during ischemia restores fine motor control and prevents neuron apoptosis and prolonged inflammation at the site of injury [28–30]. GN is known to be particularly active in models of oxidative stress and reduces accumulation of ROS following insult [31, 32]. Because of its anti-inflammatory properties, guanosine is proposed to play a role in dopamine mediation in multiple neuropathologies. In particular, GN has been shown to mitigate 6-hydroxydopamine (OHDA)-induced oxidative stress in Parkinson’s models by ameliorating mitochondrial stress and ATP depletion [32, 33]. Consequently, we hypothesize that guanosine may play a similar role in dopamine modulation during ischemic insult.

Here, we investigate guanosine’s effects on dopamine signaling in the CA1 during severe ischemia utilizing fast-scan cyclic voltammetry (FSCV). We establish DA’s patterns of release and reuptake under normoxic conditions in acute sagittal slices, then demonstrate that ischemia rapidly silences dopamine release. GN administration completely and rapidly restores DA to normoxic levels of activity. Its regulatory effect may be partially achieved through the A1 adenosine receptor, and we demonstrate that guanosine administration impacts transcription of mRNA for the dopamine transporter (DAT) and A1 and A2a receptors. Guanosine’s fast restorative activity evidenced here indicates a broad neuroprotective role in early ischemia and hints that it may function as a reuptake facilitator across diverse neurotransmitter classes. Guanosine’s dynamic protective activity suggests it is a crucial component of the brain’s native damage-mitigation response and may be a potent therapeutic target for rapid intervention strategies in ischemic stroke.

## Results

### Dopamine release is consistent in normoxia

**Fig. 1:**
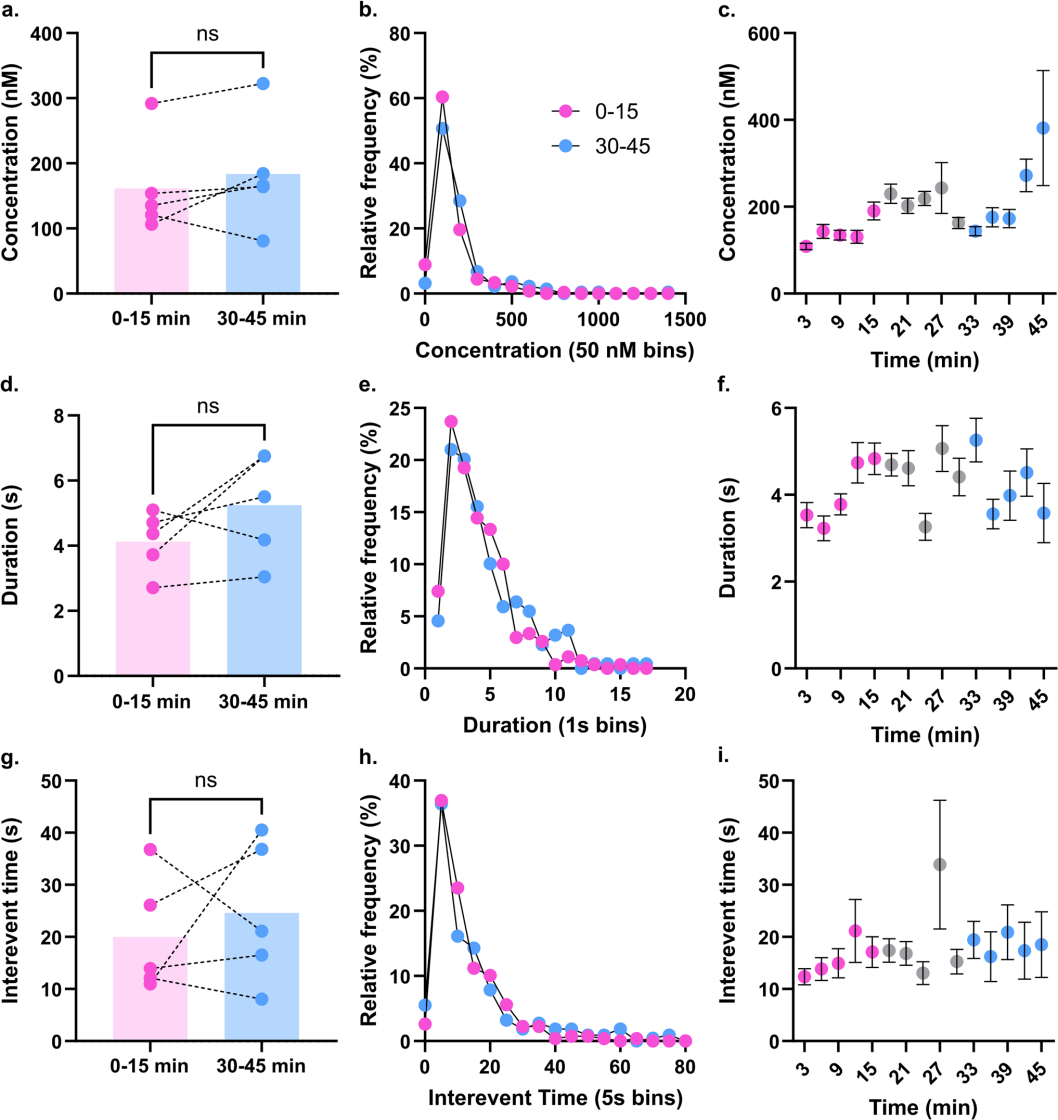
Dopamine signals consistently in the SLM under normoxic conditions. DA release was monitored with FSCV for 45 min while the brain slice was superfused with oxygenated aCSF and event concentration (**a**-**c**), duration (**d**-**f**), and interevent time (**g**-**i**) were measured. **a**) Average event concentrations per slice showing no significant changes between the first 15 and last 15 min of monitoring (Wilcoxon, *p* = 0.4375, mean_0–15_ = 161.6 ± 33.5 nM, mean_30–45_ = 183.6 ± 39.1 nM). **b**) Relative frequency of event concentrations sorted in 50 nM bins show close similarities between time regimes. **c**) Average event concentrations sorted by time in 3 min bins. **d**) Average event durations per slice show no change over time (Wilcoxon, *p* = 0.3125, mean_0–15_ = 4.12 ± 0.42 s, mean_30–45_ = 5.25 ± 0.73 s). **e**) Relative frequency of event durations sorted into 1 s bins show most events last less than 5 s for the duration of the experiment. **f**) Duration by time clustered in 3 min bins show no time-dependent trends in event length. **g**) Time between events remains consistent for 45 min of monitoring (Wilcoxon, *p* = 0.8125, mean_0–15_ = 19.97 ± 5.00 s, mean_30–45_ = 24.61 ± 6.14 s). **h**) Sorting by relative frequency for the 0–15 and 30–45 min periods show similar distributions. **i**) No trends in interevent time emerge when events are binned in 3 min periods. (*n* = 5)

Dopamine plays a complex role in the CA1, influencing glutamate modulation, spatial learning and memory, and synaptic plasticity [7, 16, 34]. It is proposed to act protectively during ischemia by mitigating glutamate-derived excitotoxicity. However, whether dopamine behaves in a net helpful or harmful manner is unclear, with conflicting literature reports showing both neuroprotective impacts and contributions to tissue damage. To evaluate dopamine’s rapid behavior under healthy conditions, we monitored DA release with FSCV for 45 min in sagittal brain slices with the electrode implanted in the *stratum lacunosum moleculare* (SLM) (Figure S1A-C) while superfusing oxygenated aCSF. This established a baseline for DA signaling patterns prior to ischemic assault and eliminated time-dependent processes as possibly confounding data collected during the treatment period of an experiment (Figure S1D). Individual DA transients were analyzed for their concentration, duration, and interevent time (IET). We established that DA is highly active in the SLM during normoxia, signaling frequently and consistently. Further, we observe no statistical differences between the first fifteen minutes of monitoring, or “control” period, and the last fifteen minutes, or “treatment” period, when solely provided oxygenated aCSF. Event concentrations remain in the low hundreds of nanomolar range for the duration of monitoring, averaging between 100–400 nM (Fig. 1A; Wilcoxon, *p* = 0.4375, mean_0–15_ = 161.6 ± 33.5 nM, mean_30–45_ = 183.6 ± 39.1 nM). Transients trend toward higher concentrations during the last five minutes of monitoring but still exhibit similar distributions of event sizes (Fig. 1B, C). Likewise, events remain in the extracellular space for 3–6 s throughout the experiment, showing no changes in reuptake rates (Fig. 1D; Wilcoxon, *p* = 0.3125, mean_0–15_ = 4.12 ± 0.42 s, mean_30–45_ = 5.25 ± 0.73 s). No trends emerged across data binned by time and event durations sorted by relative frequency showed similar distributions for both periods (Fig. 1E, F). Longer event durations were not correlated with increased concentrations, indicating that duration can be considered representative of DA movement and reuptake rather than an artifact of concentration (Figure S2; Spearman correlation matrix, *r*_C_(314) = 0.147, *p*_C_ = 0.0093). DA signaled regularly for the duration of the experiment, averaging an event every 20–25 s (Fig. 1G; Wilcoxon, *p* = 0.8125, mean_0–15_ = 19.97 ± 5.00 s, mean_30–45_ = 24.61 ± 6.14 s). As seen with both the concentration and duration datasets, the distribution of interevent times were closely matched between the first and last fifteen minutes of the experiment (Fig. 1H). No event clustering was observed; when grouped in 3-min bins, average time between events was consistent for the entirety of the experiment, with the exception of an outlier at 27 min likely attributable to biological variability (Fig. 1I). A moderate positive correlation between event duration and time until next event was observed: the longer DA spent in the extracellular space, the longer until the next event (Figure S2; Spearman correlation matrix, *r*_C_(314) = 0.498, *p*_C_ < 0.0001). This is suggestive of DA inhibitory autoregulatory behavior, likely mediated through the D2R which is present presynaptically on dopaminergic projections in the CA1 [15].

### DA signaling is suppressed in severe ischemia

**Fig. 2:**
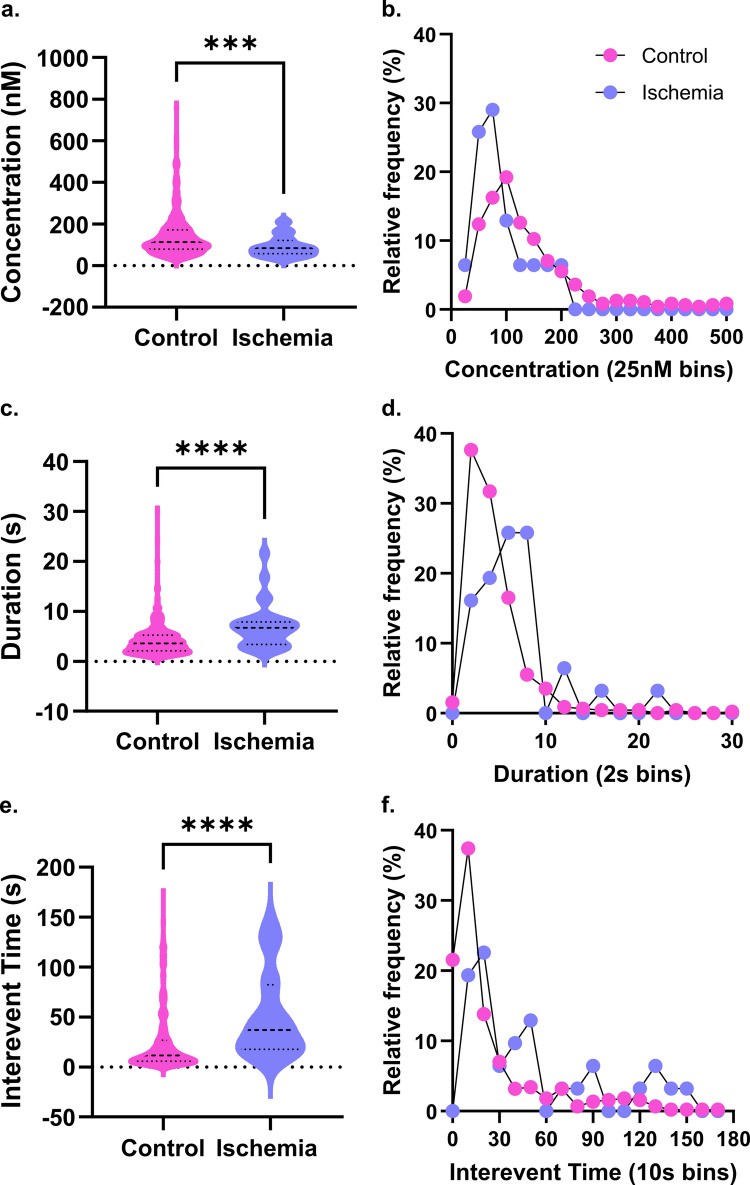
Dopamine signaling decreased during ischemic conditions. DA was monitored for 45 min; the first 15 min oxygenated aCSF was administered as a control, before switching to nitrogenated OGD for minutes 15–45. **a**) The concentration of dopamine events decreases significantly in ischemia, and **b**) event distribution shows lower median event size during OGD (two-tailed Mann–Whitney, *p* = 0.0010, mean_ctrl_ = 141.9 ± 4.7 nM, mean_isch_ = 92.5 ± 8.7 nM). **c**) DA transients spent longer in the extracellular space during ischemia and **d**) were more likely to last longer than 10 s (two-tailed Mann–Whitney, *p* < 0.0001, mean_ctrl_ = 4.4.35 ± 0.16 s, mean_isch_ = 6.82 ± 0.77 s). **e**) Average time between events increased substantially, **f**) with many events separated by 90 s or more (two-tailed Mann–Whitney, *p* < 0.0001, mean_ctrl_ = 24.73 ± 1.51 s, mean_isch_ = 50.78 ± 7.60 s). (*n* = 5)

Conversely, dopamine release drops significantly in an apparent quieting effect under severe ischemic conditions. The amount of dopamine measured per transient event decreased to under 100 nM (Fig. 2A; two-tailed Mann–Whitney, *p* = 0.0010, mean_ctrl_ = 141.9 ± 4.7 nM, mean_isch_ = 92.5 ± 8.7 nM). The distribution of event concentrations was much sharper as well, with 74.1% of transients containing ≤ 100 nM DA, compared with 49.7% in normoxic conditions (Fig. 2B). Event duration increases significantly following ischemic initiation, however, indicating extended time in the extracellular space. Mean event duration increased from 4.3 s to 6.8 s, which was likewise reflected in event distributions, which show a substantial shift towards 6–10 s events compared with the control (Fig. 2C, D; two-tailed Mann–Whitney, *p* < 0.0001, mean_ctrl_ = 4.35 ± 0.16 s, mean_isch_ = 6.82 ± 0.77 s, *n* = 5). Concomitantly, a moderate positive correlation was present between event concentration and duration, suggesting that the prolonged durations observed in ischemia may be partially attributable to an artifact of transient concentration (Figure S2; Spearman correlation matrix, *r*_I_(16) = 0.623, *p*_I_ = 0.0116, *n* = 4). However, as event concentrations decrease in ischemia, rather than increase, we consider this unlikely and instead this may reflect decreased reuptake rates for DA. The starkest change observed, however, was the quieting effect as event frequency dropped, increasing interevent time from 25 to 51 s (Fig. 2E, F; two-tailed Mann–Whitney, *p* = 0.0005, mean_ctrl_ = 24.73 ± 1.51 s, mean_isch_ = 50.78 ± 7.60 s, *n* = 5). However, the mean does not fully reflect the severity of this suppression; several slices showed a complete cessation of signaling within the 15 min incubation period. This consequently flattened the event distribution, with 16% of events spaced with at least 120 s between them. Notably, no correlation was observed between event duration and time until next event, indicating that any autoregulatory behavior had been abolished (Figure S2, Spearman correlation matrix, *r*_I_(16) = −0.094, *p*_I_ = 0.726). The low number of transients present in the ischemic cohort may obscure existing autoregulatory activity. More likely is that the severe DA dysregulation observed disrupts normal function and prevents self-regulation.

### Guanosine administration restores DA signaling

**Fig. 3:**
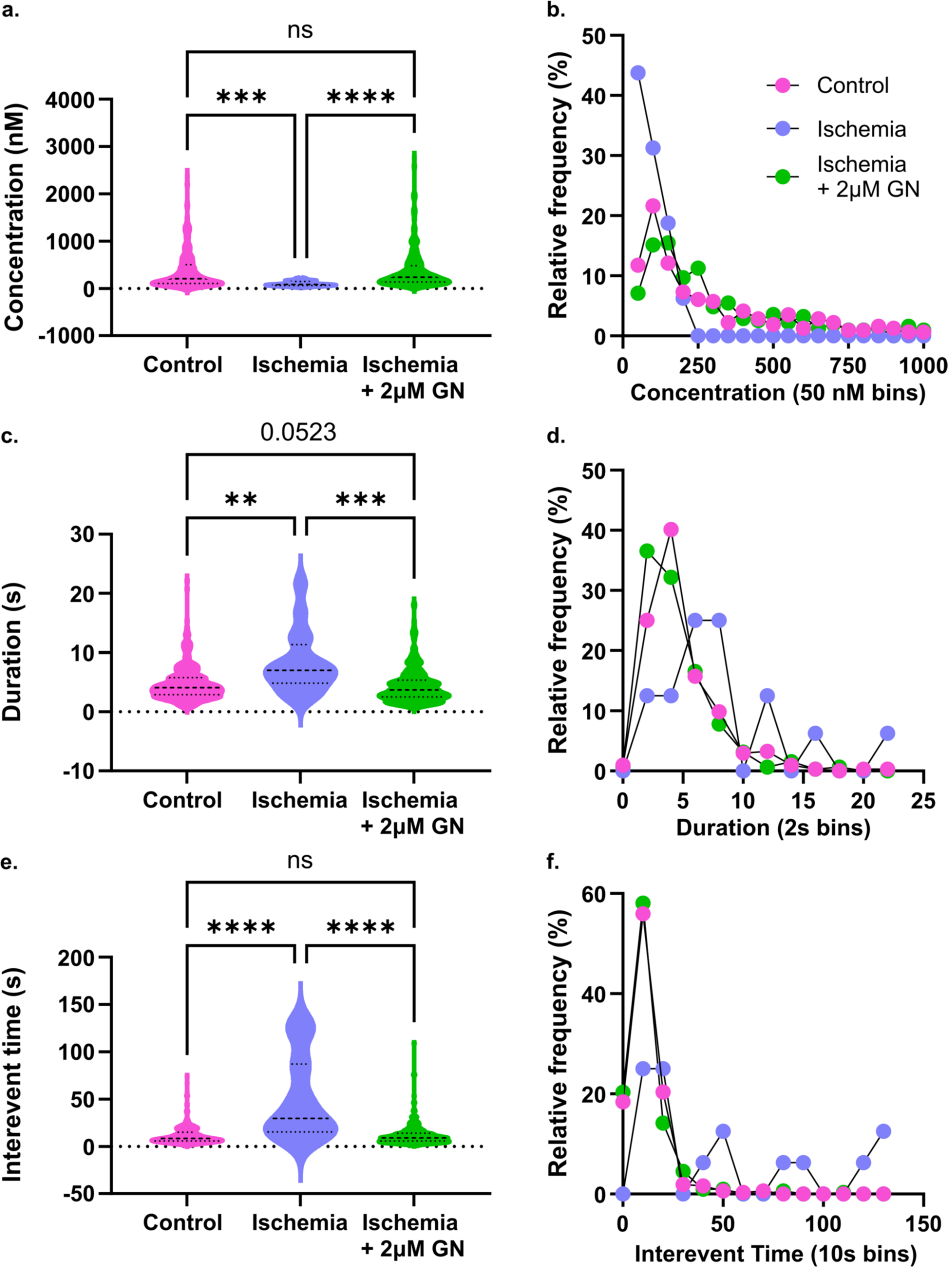
Administration of guanosine during ischemia restores DA signaling to normoxic levels. DA was monitored for 45 min, the first 15 min with oxygenated aCSF and the last 30 min with nitrogenated OGD buffer containing 2 μM guanosine. **a**) Event concentration increases with guanosine administration and is not significantly different from release levels observed in normoxic tissue, which **b**) is reflected in the similar relative frequency distributions observed between the guanosine and control datasets (Kruskal–Wallis with Dunn’s, *p*_Ctrl v Isch_ = 0.0001, *p*_Ctrl v GN_ = 0.3002, *p*_Isch v GN_ < 0.0001, mean_Ctrl_ = 370.4 ± 22.7 nM, mean_Isch_ = 99.4 ± 12.9 nM, mean_GN_ = 387.5 ± 24.3 nM). **c**) Reuptake rates are faster with GN administration and **d**) were overwhelmingly 5 s or less in duration (Kruskal–Wallis with Dunn’s, *p*_Ctrl v Isch_ = 0.0064, *p*_Ctrl v GN_ = 0.0523, *p*_Isch v GN_ < 0.0001, mean_Ctrl_ = 4.78 ± 0.17 s, mean_Isch_ = 8.23 ± 1.31 s nM, mean_GN_ = 4.35 ± 0.16 s). **e**) Time between events decreased significantly, returning signaling frequency to normoxic levels. **f**) This was reflected by the shift in event distribution back to rapid release, with 92.6% of transients occurring within 30 s of each other (Kruskal–Wallis with Dunn’s, *p*_Ctrl v Isch_ < 0.0001, *p*_Ctrl v GN_ > 0.9999, *p*_Isch v GN_ < 0.0001, mean_Ctrl_ = 11.57 ± 0.55 s, mean_Isch_ = 50.89 ± 11.00 s, mean_GN_ = 11.79 ± 0.62 s). (*n* = 5)

Guanosine release has previously been demonstrated to be upregulated within several minutes of initiation of ischemia in the CA1 [23]. Its regulatory function has previously been investigated primarily in the context of excitotoxic glutamate mediation when examined in ischemia, although mounting evidence suggests a role as a dopamine regulator. We observe that administration of a high physiological concentration of GN (2 µM) [35, 36] alongside initiation of severe ischemia completely restores DA signaling to normoxic levels compared to the previous suppression observed during oxygen–glucose deprivation. GN administration prevented the dip in concentration observed in the ischemia data set, with average event size nearing 400 nM – almost quadrupling the amount released during ischemia (Fig. 3A; Kruskal–Wallis with Dunn’s, *p*_Ctrl v Isch_ = 0.0001, *p*_Ctrl v GN_ = 0.3002, *p*_Isch v GN_ < 0.0001, mean_Ctrl_ = 370.4 ± 22.7 nM, mean_Isch_ = 99.4 ± 12.9 nM, mean_GN_ = 387.5 ± 24.3 nM, *n* = 5). Notably, the average event concentration during normoxia for this experiment was elevated above the average event concentration observed in Fig. 2A. This suggests that the amount of dopamine released varies significantly between specimens, necessitating the use of an internal control whenever possible. The event distribution was similar to the control but trended towards slightly larger transients, with an increase in median event size from 203.7 nM in normoxia to 238.4 nM with GN administration (Fig. 3B). Similarly, GN decreases average event duration from 8.2 s in ischemia to 4.4 s (Fig. 3C; Kruskal–Wallis with Dunn’s, *p*_Ctrl v Isch_ = 0.0064, *p*_Ctrl v GN_ = 0.0523, *p*_Isch v GN_ < 0.0001, mean_Ctrl_ = 4.78 ± 0.17 s, mean_Isch_ = 8.23 ± 1.31 s nM, mean_GN_ = 4.35 ± 0.16 s). This is marginally shorter than the average duration observed in the control, suggesting that GN may be facilitating reuptake in addition to restoring DA release. This was further validated in the frequency distribution; the GN data set was shifted slightly towards lower durations, with events 2 s or shorter accounting for 37.2% compared to 26.0% in normoxia (Fig. 3D). We did not observe any correlation between transient concentration and duration, indicating the two data sets are not convoluted (Figure S2). Most significantly, GN administration restores event frequency to rates observed in healthy tissue (Fig. 3E; Kruskal–Wallis with Dunn’s, *p*_Ctrl v Isch_ < 0.0001, *p*_Ctrl v GN_ > 0.9999, *p*_Isch v GN_ < 0.0001, mean_Ctrl_ = 11.57 ± 0.55 s, mean_Isch_ = 50.89 ± 11.00 s, mean_GN_ = 11.79 ± 0.62 s). Whereas ischemia near-completely abolishes DA release absent treatment, GN intervention prevents DA stoppage and maintains consistently high signaling rates for the entirety of the monitored period. The distribution of interevent times is near-identical to that observed in normoxic conditions (Fig. 2F). This is further reflected in the restoration of the duration-time to next event correlation observed under normal conditions, suggesting that frequent signaling is essential to the suggested autoregulatory behavior (Figure S2; Spearman correlation matrix, *r*_I+G_(310) = 0.500, *p*_I+G_ =  < 0.0001).

### GN effects may occur through adenosine receptors and DAT

**Fig. 4:**
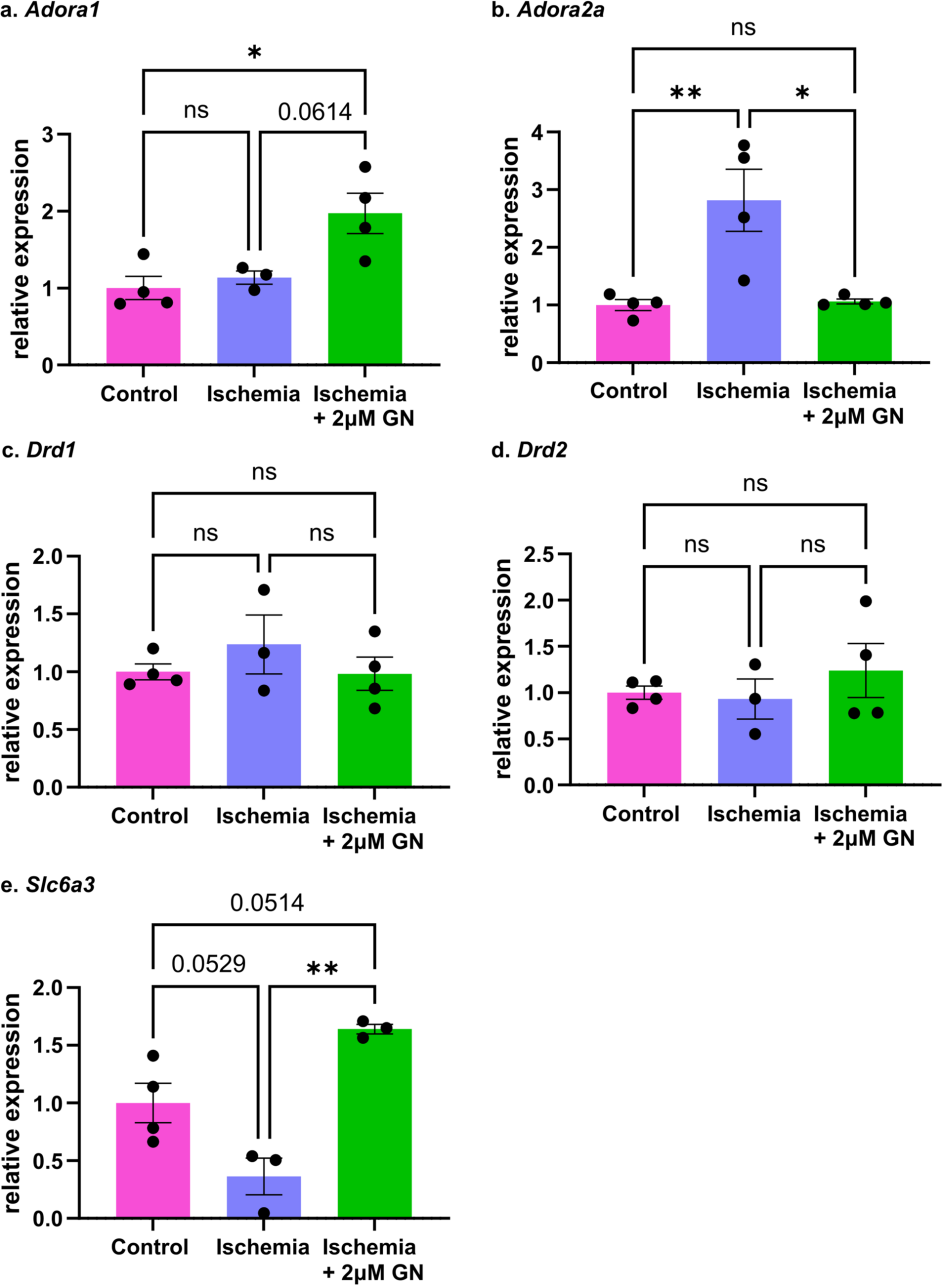
Guanosine’s fast protective effects on dopamine may be mainly propagated through adenosine receptors and the dopamine transporter. **a**) *Adora1* is upregulated by GN administration in ischemia (ANOVA with Bonferroni, *p*_Ctrl v Isch_ > 0.9999, *p*_Ctrl v GN_ = 0.0203, *p*_Isch v GN_ = 0.0614, mean_Ctrl_ = 1.00 ± 0.15-fold change, mean_Isch_ = 1.14 ± 0.08 -fold change, mean_GN_ = 1.97 ± 0.26-fold change). **b**) *Adora2a* is upregulated in ischemia but lowered to normoxic levels of transcription with GN administration (ANOVA with Bonferroni, *p*_Ctrl v Isch_ = 0.0085, *p*_Ctrl v GN_ > 0.9999, *p*_Isch v GN_ = 0.0105, mean_Ctrl_ = 1.00 ± 0.10-fold change, mean_Isch_ = 2.82 ± 0.45 -fold change, mean_GN_ = 1.06 ± 0.04-fold change). **c**) Transcription of *Drd1* does not significantly change in ischemia or with GN treatment (ANOVA with Bonferroni, *p*_Ctrl v Isch_ = 0.9645, *p*_Ctrl v GN_ > 0.9999, *p*_Isch v GN_ = 0.8704, mean_Ctrl_ = 1.00 ± 0.07-fold change, mean_Isch_ = 1.24 ± 0.25 -fold change, mean_GN_ = 0.98 ± 0.14-fold change). **d**) Similarly, no changes in *Drd2* transcription were evident (ANOVA with Bonferroni, *p*_Ctrl v Isch_ > 0.9999, *p*_Ctrl v GN_ > 0.9999, *p*_Isch v GN_ > 0.9999, mean_Ctrl_ = 1.00 ± 0.07-fold change, mean_Isch_ = 0.93 ± 0.22 -fold change, mean_GN_ = 1.24 ± 0.29-fold change). **e**) Expression of *Slc6a3* trends downward in ischemia but is significantly upregulated with GN administration (ANOVA with Bonferroni, *p*_Ctrl v Isch_ = 0.0529, *p*_Ctrl v GN_ = 0.0514, *p*_Isch v GN_ = 0.0020, mean_Ctrl_ = 1.00 ± 0.17-fold change, mean_Isch_ = 0.36 ± 0.16 -fold change, mean_GN_ = 1.64 ± 0.04-fold change) (*n* = 4)

Analysis of hippocampal mRNA profiles reveals that GN administration has early impacts on transcription for adenosine receptors A1R and A2aR and the dopamine transporter DAT. Intact slices were incubated in normoxia, ischemia, or ischemia + 2 μM GN before sectioning out the hippocampus and isolating RNA. Tissue was analyzed for changes in *Adora1* and *Adora2a*, corresponding to the adenosine A1 and A2A receptors, *Drd1* and *Drd2*, corresponding to the dopamine D1 and D2 receptors, and *Slc6a3*, corresponding to the dopamine transporter DAT. *Adora1* is not upregulated in ischemia but doubles with GN addition, while an inverse relationship is observed for *Adora2a*: ischemia triples transcription, while GN lowers transcription to levels observed in normoxic conditions (Fig. 4A, B; ANOVA with Bonferroni, *Adora1*: *p*_Ctrl v Isch_ > 0.9999, *p*_Ctrl v GN_ = 0.0203, *p*_Isch v GN_ = 0.0614, mean_Ctrl_ = 1.00 ± 0.15-fold change, mean_Isch_ = 1.14 ± 0.08 -fold change, mean_GN_ = 1.97 ± 0.26-fold change; *Adora2a*: *p*_Ctrl v Isch_ = 0.0085, *p*_Ctrl v GN_ > 0.9999, *p*_Isch v GN_ = 0.0105, mean_Ctrl_ = 1.00 ± 0.10-fold change, mean_Isch_ = 2.82 ± 0.45 -fold change, mean_GN_ = 1.06 ± 0.04-fold change, *n* = 4). No significant changes were observed in transcription of *Drd1* and *Drd2* in ischemia or with guanosine administration (Fig. 4C, D; *Drd1*: ANOVA with Bonferroni, *p*_Ctrl v Isch_ = 0.9645, *p*_Ctrl v GN_ > 0.9999, *p*_Isch v GN_ = 0.8704, mean_Ctrl_ = 1.00 ± 0.07-fold change, mean_Isch_ = 1.24 ± 0.25 -fold change, mean_GN_ = 0.98 ± 0.14-fold change; *Drd2*: *p*_Ctrl v Isch_ > 0.9999, *p*_Ctrl v GN_ > 0.9999, *p*_Isch v GN_ > 0.9999, mean_Ctrl_ = 1.00 ± 0.07-fold change, mean_Isch_ = 0.93 ± 0.22 -fold change, mean_GN_ = 1.24 ± 0.29-fold change, *n* = 4). Ischemic conditions suppressed *Slc6a3* transcription to a third of its normoxic levels; however, GN administration increases production five-fold over ischemia and by 60% compared to normoxia (Fig. 4E; ANOVA with Bonferroni, *p*_Ctrl v Isch_ = 0.0529, *p*_Ctrl v GN_ = 0.0514, *p*_Isch v GN_ = 0.0020, mean_Ctrl_ = 1.00 ± 0.17-fold change, mean_Isch_ = 0.36 ± 0.16 -fold change, mean_GN_ = 1.64 ± 0.04-fold change, *n* = 4).

## Discussion

Guanosine’s role as a neuroprotector in ischemia has primarily been defined by its involvement in glutamate reuptake and mitigation of NMDAR-induced excitotoxicity within several hours to days following stroke [24, 30, 37]. Its downstream effects are well-established; it reduces oxidative stress, promotes anti-inflammatory cytokine production, mitigates tissue infarction, and decreases morbidity and loss of motor function [30, 38, 39]. However, this presents two clear gaps in our current understanding of GN activity: no analysis of guanosine’s immediate role in neuroprotection during an ischemic attack has been reported, and our purview of its contributions are narrowly limited to one neurotransmitter. While glutamate is the most prominent player in ischemic damage in the CA1, it is far from the only neurochemical – or even the only neurotransmitter – dynamically responding to injury. Previous work from our lab indicates that GN signaling is immediately upregulated following initiation of ischemia, suggesting a role in seconds- to minutes-scale regulatory activity [23]. With this in mind, we investigate GN’s role in real-time modulation of dopamine release in the CA1 immediately following ischemic initiation.

Here, we present the first real-time investigation of dopamine dynamics in the SLM. Dopamine signaling patterns have been well established in the striatum, but have not been well-investigated in the CA1 despite innervation from the VTA and CA3 which contribute to learning, long-term potentiation, and memory development [7–9]. We establish here that dopamine signals at low concentrations but with high frequency in the SLM. The release pattern established is distinct from those reported in other regions; the average quantity of dopamine released was lower than observed in the caudate putamen, but was far more frequent [40]. Similarly, signaling frequency was higher in the CA1 than in the dorsal striatum or nucleus accumbens shell or core [41, 42]. This may be partially attributable to the use of males in this study, as previous work has found increased DA release in males over females [41]. As the study was performed ex vivo measuring spontaneous release, this indicates that dopamine’s activity in the CA1 is not limited to learning-evoked stimuli. Dopamine is considered a neuroregulator as well as a neurotransmitter; it modulates glutamate effects in memory development through D1R and D5R association with and suppression of NMDAR activity [7, 17, 43]. Likewise, D2R is neuroprotective against glutamate excitotoxicity in the CA1 and CA3 and can protect against kainate-induced seizures [44]. It stands to reason, therefore, that maintaining DA activity in ischemia will induce protective effects and makes it a potential target for purinergic regulation.

Dopamine’s subsequent downregulation in severe ischemic conditions absent any therapeutic strategy presents a significant opening for neuroprotective intervention. We observe a near-complete elimination of DA release following initiation of ischemia. The few transient events that do occur are markedly longer, indicating substantial slowing of reuptake. Slowed reuptake may cause increased DA accumulation in the extracellular space to maintain its protective effect, but the loss of consistent dopamine signaling suggests that dopamine dysregulation may contribute to glutamate excitotoxicity, as NMDAR activity is no longer suppressed. As we use a model of severe global ischemia here, it is possible that the degree of DA quieting observed is influenced by damage occurring in the VTA and CA3 as well. Previously our lab demonstrated that on-chip delivery of focal ischemia to the caudate putamen increased DA concentrations, suggesting that locally contained attacks in the striatum likely do not lose the neuroprotective effects of dopamine [45]. Instead, the quieting effect we observe may be dependent on processes initiated from ischemic damage outside of the CA1. Whereas focal damage only initiates a local protective response, the severity of global ischemia induces more neurotoxic conditions, which may contribute to the observed decrease in dopamine as neurons sustain damage.

Guanosine administration completely restores DA signaling to healthy patterns despite sustained ischemic conditions. We utilized 2 μM GN, a much lower concentration than utilized by previous models of guanosine-based neuroprotection, which is more representative of accumulated levels within 18–24 h following ischemic insult [35, 37, 46]. Notably, variation within average DA event concentration between specimens during normoxia suggests that endogenous DA release in the CA1 is more variable than the highly consistent concentrations observed from evoked DA in more richly innervated regions [41]. Consequently, although the overall mean DA concentration in normoxic conditions is lower than the average concentration measured during ischemia + GN treatment, internal comparison within samples reveals that DA is maintained at preischemic levels, rather than increased.

GN administration prevents cessation of DA signaling and maintains high activity for the duration of ischemia, indicating a previously unidentified role as a rapid dopamine regulator. This role may be partially achieved through facilitation of dopamine reuptake. DA event duration trends towards shorter periods in the extracellular space compared to normoxic events, potentially indicating faster transport from the extracellular space. This is further supported by GN’s upregulation of DAT mRNA *Slc6a3*, which sees a five-fold increase in expression compared to its downregulation in ischemia absent treatment. Few studies have examined changes in transporter transcription levels immediately following short ischemic events. However, decreased *Slc6a3* expression has similarly been observed in longer middle cerebral artery occlusion (MCAO) models [47]. Short-term changes in the transcriptome following ischemia have not been well characterized; however, DAT expression is regulated by transcriptional factors, such as Nurr1, which are susceptible to changes in the MAPK and PI3K pathways during ischemia [48–50] DAT’s role in ischemia is controversial, and DAT antagonism has been proposed as a potential therapeutic avenue for treatment of stroke [47]. We propose that the guanosine-based upregulation observed here may play several roles. First, GN is ubiquitously engaged in maintaining homeostasis across a wide range of pathologies, and increased DAT expression may reflect a reversal of damage-inflicted downregulation. Second, increased DAT availability results in faster clearance and therefore increased recycling and repackaging of dopamine. DAT is also a primary mechanism for regulating dopamine activity. Particularly given the presence of presynaptic D2Rs that serve as inhibitory autoreceptors, faster DA clearance can prevent self-inflicted downregulation and thereby elimination of its regulatory effects. Bidirectional dopamine transport independent of vesicular release has also been reported and may represent a potential pathway for modulatory dopamine response to ischemic stress [51, 52]. Further, guanosine may play a more direct role in modulating DAT expression; GN is known to promote expression of glutamate transporters through the PI3K/Akt and MAP kinase pathways in ischemia, which also influence expression of DAT [53]. However, this territory is unexplored and further investigation of the mechanistic relationship between guanosine, DAT, and ischemia is necessary.

In addition to regulating transport, guanosine administration impacts dopamine release in a response potentially modulated through the adenosine A1 and A2a receptors, as well as the dopamine transporter DAT. Guanosine is considered an “orphan neuromodulator”; the existence of a putative GPCR guanosine receptor has been verified but not identified [38, 54]. Adenosine receptors are also expressed densely and ubiquitously, as they play an essential role in maintaining homeostasis and modulating neuron-microglial communication [55, 56]. Consequently, guanosine’s neuroprotective activity has primarily been investigated through its association at the adenosine A1 and A2A receptors. Many of GN’s downstream impacts, including reduction of ROS accumulation and activation of the PI3K/Akt pathway, are achieved through the A1R [25, 32, 33, 57].

We do not see evidence of upregulation of *Drd1* or *Drd2* after exposure to ischemia, with or without GN. This suggests that guanosine’s regulatory activities may be directed more broadly, rather than specifically targeting dopamine receptors. However, definitive changes were observed in expression of both *Adora1* and *Adora2a* in opposite directions: *Adora1* was exclusively upregulated with guanosine addition, whereas *Adora2a* was elevated during ischemia but reduced to normoxic levels following GN administration. Guanosine has previously been demonstrated to increase extracellular adenosine levels, and given its known activity at A1R, this serves both as a downstream neuroprotective effect and a direct upregulation of guanosine’s own protective ability [58]. Conversely, the A2AR can have injurious effects following ischemic damage as it prevents the D2R from suppressing NMDAR activity – and thus glutamate excitotoxicity [8, 59]. Guanosine’s suppression of A2AR synthesis, therefore, may represent an avenue through which D2R-mediated neuroprotection is enabled.

## Conclusion

Here, we demonstrate for the first time that guanosine can effectively intervene in dopamine dysregulation during ischemia. We establish that DA signaling in the SLM is suppressed within several minutes following initiation of severe global ischemia but can be fully restored with administration of low concentrations of guanosine. We also observe guanosine-induced changes in transcription of mRNA for DAT, A1R, and A2aR, suggesting that dopamine reuptake is facilitated by GN as well. This broadens our understanding of guanosine in ischemia from a glutamate-specific perspective to a potential role as a promoter of multi-class neurotransmitter reuptake. Although guanosine is often ignored in favor of adenosine- and ATP-based purinergic regulation, we provide significant evidence that its importance in neuroprotection during ischemia cannot – and should not – be overlooked, but rather considered an essential part of purine-based modulation.

## Materials & methods

An extended materials & methods is detailed in the supplement; all information regarding experiment design is described below.

### Tissue preparation

Prior to experiments, rats were anesthetized via isoflurane inhalation then immediately euthanized via decapitation. The brain was extracted whole and placed in oxygenated (95% O_2_, 5% CO_2_) ice-cold aCSF for 1 min. Each hemisphere was mounted on a vibratome stage and secured with super glue for slicing. A Leica VT1000S vibratome (Chicago, IL, USA) was used to obtain sagittal 400 μm-thick hippocampal slices at a frequency of 3 and speed of 90. After slicing, tissue recovered at room temperature in oxygenated aCSF for at least 1 h before experimentation.

### Fast scan cyclic voltammetry

For voltammetric experiments, individual slices were placed in a standard superfusion chamber (Warner Instruments, Hamden, CT, USA) superfused with oxygenated aCSF at 2 mL min^−1^ between 34–37 °C via a Watson-Marlow 205S peristaltic pump (Wilmington, MA, USA). A carbon fiber microelectrode was implanted approximately 75 μm into the *stratum lacunosum-moleculare* in the CA1 region of the dorsal hippocampus via a Narishige MM-3 micromanipulator. The electrode was equilibrated for 5 min using the dopamine waveform described above at an application frequency of 60 Hz before switching to the collection frequency of 10 Hz. FSCV data was collected continuously for 45 min per slice. For the first 15 min of data collection, the slice was provided exclusively oxygenated aCSF; this was immediately followed by 30 min of buffer for the appropriate experimental condition (e.g., nitrogenated (100% N_2_) OGD buffer for ischemia) (Figure S2D). Transient DA signaling was identified from a false color plot (Figure S1A) and through characteristic oxidation and reduction on cyclic voltammograms (Figure S1B). For each transient event, concentration, duration, and interevent time are reported. Concentration is defined as the maximum concentration measured and was determined from a concentration curve calibrated on the potentiostat used for data collection (Figure S3); duration is defined as Δ*t* at half-max current; interevent time is calculated from the time between transient peaks.

### RNA isolation and real-time quantitative PCR

Total RNA was extracted from the CA1 region of the hippocampus using the Thermo Fisher PureLink™ RNA Mini Kit, following the manufacturer's protocol with minor adjustments in 1–2 steps, and quantified with a NanoDrop spectrophotometer. Primers were designed and validated using NCBI Primer-BLAST, purchased from Integrated DNA Technologies as RxnReady Oligos, and diluted to a 1 µM stock solution. mRNA expression was quantified by qRT-PCR using the Bio-Rad iTaq™ Universal SYBR® Green One-Step RT-PCR Kit, with 100 nM primer and 150 ng of total RNA in a 10 µL reaction. PCR products were verified by melting temperature. mRNA expression levels relative to Gapdh were normalized to normoxia-treated controls.

## Supplementary Information

Below is the link to the electronic supplementary material.

Supplementary file1 (DOCX 418 KB)

## Data Availability

Data is provided within the manuscript or supplementary information files.
