# Supplementary material for: Guanosine contributes to rapid purinergic regulation of dopamine during ischemia
Source: Purinergic Signal. 2026 Apr 20;22(3):41. doi: 10.1007/s11302-026-10153-7 (PMC13096455; doi:10.1007/s11302-026-10153-7)
Supplement: Supplementary file 1 — Supplementary file1 (DOCX 418 KB) [file 11302_2026_10153_MOESM1_ESM.docx]

**Supporting Information for**

Guanosine Contributes to Rapid Purinergic Regulation of Dopamine during Ischemia

Moriah E. Weese-Myers^1^, Alaa S. Abdelrazeq Hassan^1^, Ashley E. Ross^1,^*

*^1^University of Cincinnati, Cincinnati OH, USA 45221*

Correspondence to:

Department of Chemistry

University of Cincinnati

312 College Dr.

404 Crosley Tower

Cincinnati, OH USA 45221-0172

Email: [ross2ah@ucmail.uc.edu](mailto:ross2ah@ucmail.uc.edu)

* corresponding author

**This PDF file includes:**

Extended Materials & Methods

Figures S1 to S3

**Extended Materials and Methods**

*Chemicals & Reagents*

Except where otherwise specified, all reagents were purchased from Fisher Scientific (Fair Lawn, NJ, USA). All aqueous solutions were made using Milli-Q deionized water (Millipore, Billerica, MA). All brain slice experiments were conducted in artificial cerebrospinal fluid (aCSF) or oxygen-glucose deprivation buffer (OGD). Guanosine (GN) were added to OGD buffer where noted. Normoxic aCSF was prepared daily and contained 2.5 mM KCl, 1.2 mM NaH2PO4, 2.4 mM CaCl2, 1.2 mM MgCl2, 126 mM NaCl, 11 mM D-glucose, 4 mM sucrose, 25 mM NaHCO3, and 15 mM tris(hydroxymethyl)aminomethane. Normoxic aCSF was oxygenated (95% O_2_, 5% CO_2_) during slice preparation and experiments. All ischemia experiments were conducted in OGD, which contained the same components as aCSF excepting D-glucose and sucrose. OGD was deoxygenated with N_2_ gas for the duration of ischemia delivery. For guanosine delivery experiments, GN was prepared as a 10 mM stock solution in 0.1 M HCl and added to OGD buffer for a final concentration of 2 μM on the day of use.

*Carbon Fiber Microelectrode Fabrication*

Carbon fiber microelectrodes (CFMEs) were home fabricated from 7 μm T-650 PAN-based carbon fibers (Mitsubishi Chemical Carbon Fiber and Composites, Sacramento, CA, USA). Fibers were aspirated into 1.2 × 0.68 mm glass capillaries (A&M Systems, Sequim, WA, USA) and pulled in two with a vertical magnetic PE-22 Electrode Puller (Narishige, Tokyo, Japan), forming a glass seal. Electrodes were manually trimmed under a microscope with a scalpel 100-150 μm from the glass seal. Prior to use, electrodes were backfilled with 1M KCl supporting electrolyte.

*Electrochemical Set-Up*

Cyclic voltammograms were collected with a Dagan Chem-Clamp 5-MEG potentiostat (Dagan Corp, Minneapolis, MN, USA) coupled to a UNC breakout box (UNC Electronics Shop, Chapel Hill, NC, USA). Data acquisition and analysis were performed using high-definition cyclic voltammetry (HDCV) software (UNC at Chapel Hill) paired with a multifunction I/O device (PC1e-6363, National Instruments, Austin, TX, USA). Data were collected using a triangular waveform scanning from -0.4 V to 1.3 V and back at a scan rate of 400 V/s and an application frequency of 10 Hz. All data were passed through a 3 kHz low-pass filter. Voltammograms were background subtracted to remove capacitive current prior to analysis. Dopamine event concentration was calculated from *i_p_* at 0.7 V utilizing the slope of our calibration curve (Figure S1).

*Animal Use*

All animal use was approved by the Institutional Animal Care and Use Committee (IACUC) at the University of Cincinnati and was in accordance with the National Research Council’s guidelines in *The Guide for the Care and Use of Laboratory Animals* (“*The Guide*”). Male adolescent Sprague-Dawley rats between 170-180g (Charles River Laboratories, Wilmington, MA, USA) were housed in a university vivarium accredited by the Association for Assessment and Accreditation of Laboratory Animal Care (AAALAC). Rats were housed in pairs, provided food and water *ad libitum*, and kept on a 12 h light/dark cycle.

*RT-qPCR Primers*

| Sequence-based reagent Primers | Forward: CTCCGTGGGACCAATGTCTT  Reversed: CAGTGCCCATGCGATGATGA | Integrated DNA Technologies | SLC6a3 |
| --- | --- | --- | --- |
| Sequence-based reagent Primers | Forward: GTGAAGGTGAACCAGGCACT  Reversed: ACACTCAGGTTGTTCCAGCC | Integrated DNA Technologies | Adora1 |
| Sequence-based reagent Primers | Forward: CTCGTGGCTCTTGTGAGGAA  Reversed: AGGACCAGGACAAAACAGGC | Integrated DNA Technologies | Adora2 |
| Sequence-based reagent Primers | Forward: TCTCCTTTCGCATCCTCACG Reversed: AAATTGCCATCCAAGGGCCA | Integrated DNA Technologies | Drd1 |
| Sequence-based reagent Primers | Forward: TGACGATCTGGAGAGGCAGA  Reversed: GATGGCACACAGGTTCAGGA | Integrated DNA Technologies | Drd2 |
| Sequence-based reagent Primers | Forward: TTGCCATCAACGACCCCTTC  Reversed: TAAGCAGTTGGTGGTGCAGG | Integrated DNA Technologies | Gapdh  (Housekeeping) |

*Statistics*

All statistical analyses were performed in GraphPad Prism v. 10.4 (GraphPad Software, La Jolla, CA, USA). Results were considered statistically significant at p < 0.05 (95% confidence level). Unless specified, data are reported as the mean ± the standard error of the mean (SEM). For FSCV, TTC, and immunohistochemistry data, *n* represents the number of brain slices. For RT-qPCR, *n* represents the number of animals. Data normality was determined via QQ plot and Shapiro-Wilk for all data sets. All comparison analyses are two-tailed.


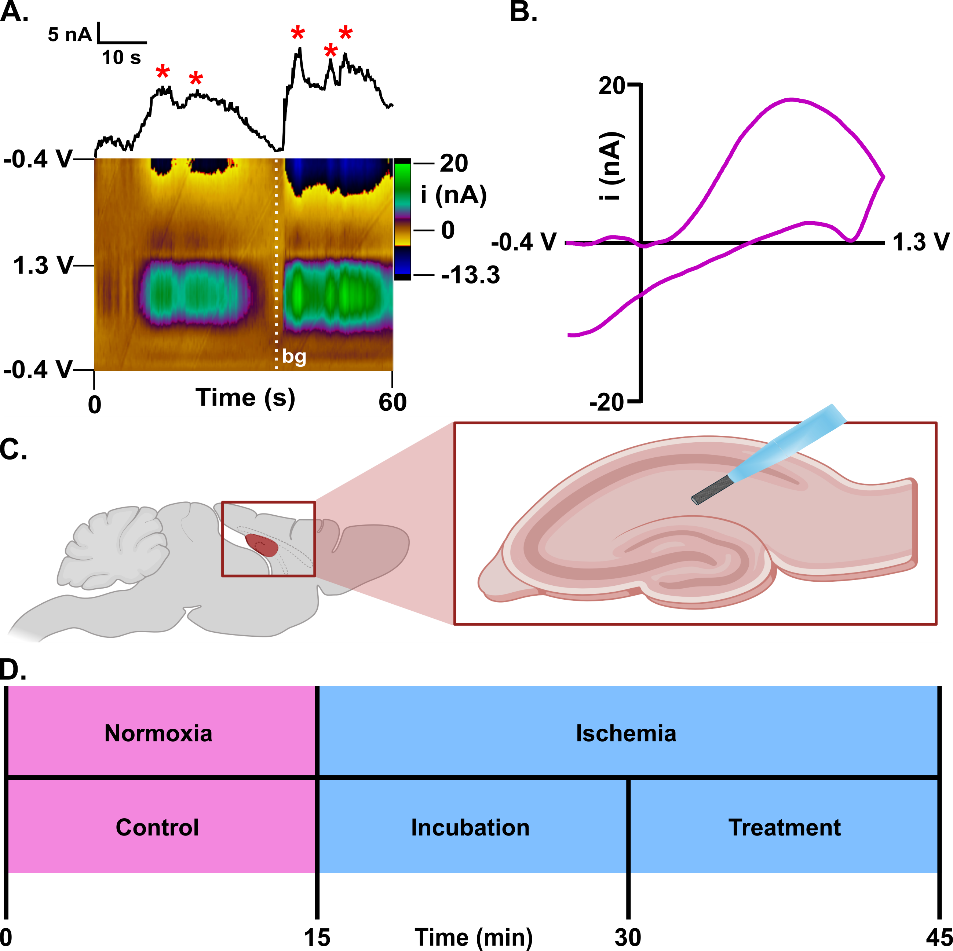

**Fig. S1** Graphical depiction of methods employed for DA monitoring. A) Dopamine transients are measured using FSCV and transients are identified from a false color plot and current vs. time trace. B) Dopamine’s identity is confirmed from its cyclic voltammogram, or “electrochemical fingerprint.” C) A carbon fiber microelectrode is implanted in the SLM in the CA1 of an acute sagittal brain slice. D) All FSCV experiments follow the same scheme: 15 minute superfusion of normoxic aCSF followed by 30 minutes of superfused treatment buffer; data are compared from the first and last 15 minutes of the experiment.

**
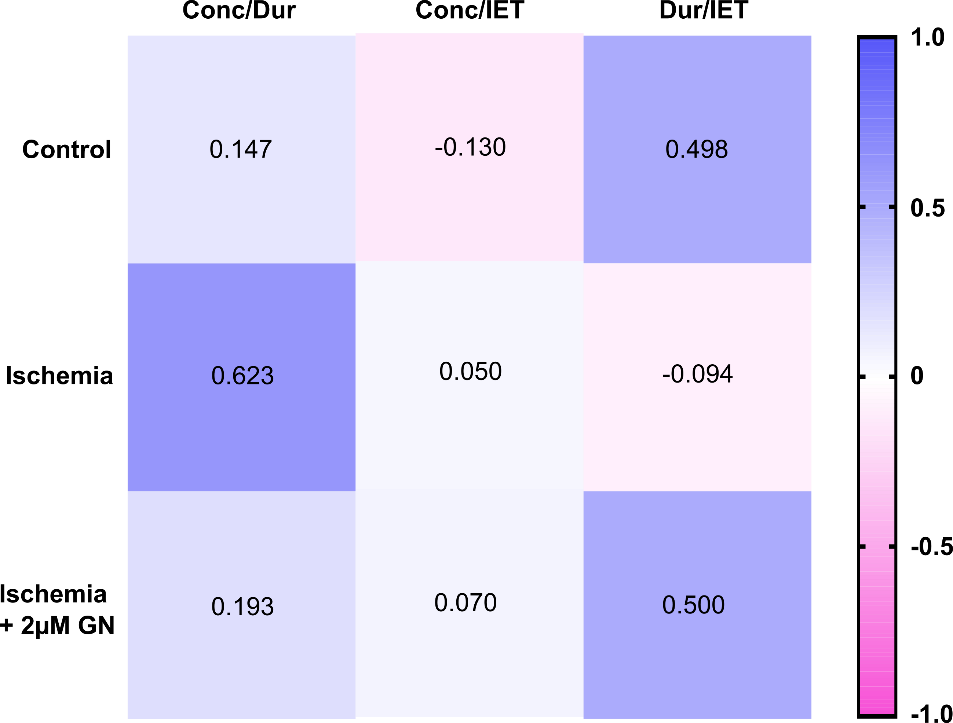
**

**Fig. S2** Spearman correlations were performed between event concentration, duration, and interevent time for all dopamine transients in the control (Fig. 2), ischemia (Fig. S2), and guanosine addition (Fig. 3) studies. A moderate positive correlation between concentration and duration was observed solely in the ischemia cohort (*r*_C_(314) = 0.147, *p*_C_ = 0.0093; *r*_I_(16) = 0.623, *p*_I_ = 0.0116; *r*_I+G_(310) = 0.193, *p*_I+G_ = 0.0006). No relationship was observed between concentration and time until next event (*r*_C_(314) = -0.130, *p*_C_ = 0.0269; *r*_I_(16) = 0.050, *p*_I_ = 0.8541; *r*_I+G_(310) = 0.070, *p*_I+G_ = 0.2246). Moderate positive correlations were observed between event duration and interevent time for the control and guanosine groups, but not the ischemia group (*r*_C_(314) = 0.498, *p*_C_ < 0.0001; *r*_I_(16) = -0.094, *p*_I_ = 0.726; *r*_I+G_(310) = 0.500, *p*_I+G_ = < 0.0001)


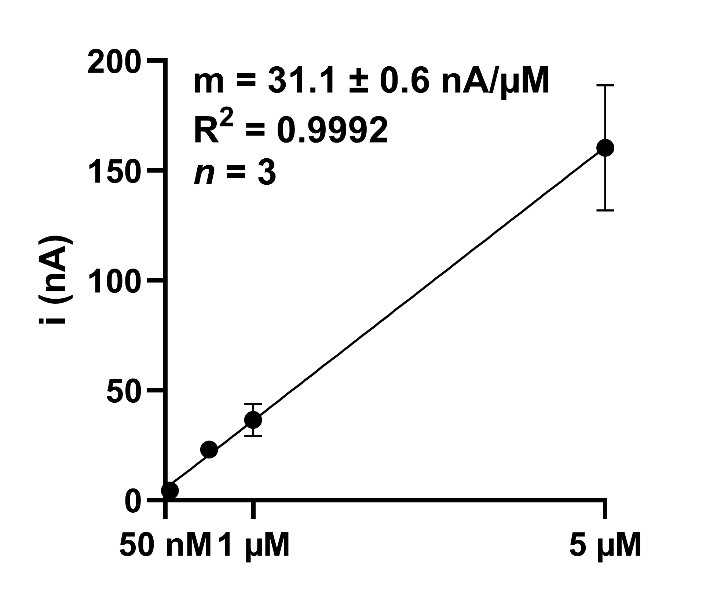


Fig. S3 Concentration curve for dopamine spanning a working range of 50 nM-5 µM. The sensitivity is 31.1 ± 0.6 nA/µM and the limit of detection is 1.5 ± 0.2 nM with a high goodness of fit (R^2^ = 0.9992, *n* = 3)
